# Poly Lactic-co-Glycolic Acid (PLGA) Loaded with a Squaraine Dye as Photosensitizer for Antimicrobial Photodynamic Therapy

**DOI:** 10.3390/polym16141962

**Published:** 2024-07-09

**Authors:** Degnet Melese Dereje, Carlotta Pontremoli, Ana García, Simone Galliano, Montserrat Colilla, Blanca González, María Vallet-Regí, Isabel Izquierdo-Barba, Nadia Barbero

**Affiliations:** 1 Department of Chemistry, NIS Interdepartmental and INSTM Reference Centre, University of Torino, Via G. Quarello 15A, 10135 Torino, Italy; degnetmelese.dereje@unito.it (D.M.D.); carlotta.pontremoli@unito.it (C.P.); simone.galliano@unito.it (S.G.); 2 Department of Chemical Engineering, Bahir Dar Institute of Technology, Bahir Dar University, Polypeda 01, Bahir Dar 0026, Ethiopia; 3 Department of Chemistry in Pharmaceutical Sciences, Faculty of Pharmacy, Universidad Complutense de Madrid, Research Institute Hospital 12 de Octubre (i+12), Plaza Ramón y Cajal s/n, 28040 Madrid, Spain; anagfontecha@ucm.es (A.G.); mcolilla@ucm.es (M.C.); blancaortiz@ucm.es (B.G.); vallet@ucm.es (M.V.-R.); 4 Networking Research Centre on Bioengineering, Biomaterials and Nanomedicine (CIBER-BBN), Spain; 5 Institute of Science and Technology for Ceramics (ISSMC-CNR), Via Granarolo, 64, 48018 Faenza, Italy

**Keywords:** Antimicrobial Photodynamic Therapy, squaraine dyes, PLGA nanoparticles, designs of experiments, Gram-positive bacteria

## Abstract

Antimicrobial Photodynamic Therapy (aPDT) is an innovative and promising method for combating infections, reducing the risk of antimicrobial resistance compared to traditional antibiotics. Squaraine (SQ) dyes can be considered promising photosensitizers (PSs) but are generally hydrophobic molecules that can self-aggregate under physiological conditions. To overcome these drawbacks, a possible solution is to incorporate SQs inside nanoparticles (NPs). The present work deals with the design and development of innovative nanophotosensitizers based on poly lactic-co-glycolic acid (PLGA) NPs incorporating a brominated squaraine (BrSQ) with potential application in aPDT. Two designs of experiments (DoEs) based on the single emulsion and nanoprecipitation methods were set up to investigate how different variables (type of solvent, solvent ratio, concentration of PLGA, stabilizer and dye, sonication power and time) can affect the size, zeta (ζ)-potential, yield, entrapment efficiency, and drug loading capacity of the SQ-PLGA NPs. SQ-PLGA NPs were characterized by NTA, FE-SEM, and UV-Vis spectroscopy and the ability to produce reactive oxygen species (ROS) was evaluated, proving that ROS generation ability is preserved in SQ-PLGA. In vitro antimicrobial activity against Gram-positive bacteria in planktonic state using *Staphylococcus aureus* was conducted in different conditions and pH to evaluate the potential of these nanophotosensitizers for aPDT in the local treatment of infections.

## 1. Introduction

Over the past two decades, researchers have observed that the number of multidrug-resistant pathogenic microorganisms or complex architectures (biofilms) has gradually increased [1,2,3], making them subsequently highly recalcitrant to antibiotic therapies and immune clearance. On the other hand, the approval of new antibiotics has drastically slowed down, generating a major global health problem [4]. The increasing resistance response of pathogenic microorganisms to commonly used antibiotics along with their inappropriate use has led to new resistance mutations and slows down the treatment process of common infectious diseases [5,6]. According to the UN 2023 report, which aims to strengthen environmental action in response to antimicrobial resistance, the global failure to tackle the problem of antibiotic resistance will cause up to 10 million deaths and an estimated cost of £66 trillion by 2050 [7]. The end of the “antibiotic era” paves the way for the development of innovative alternative approaches that show highly destructive microbial pathogens, overcoming the resistance mechanism [8,9,10]. Currently, nanotechnology offers promising strategies in combating bacterial infections through the design of stimuli-responsive nanosystems to improve the therapeutic efficacy [11]. In this context, light is a very advantageous stimulus because it brings up the possibility to engineer innovative nanoplatforms for combined therapies [12,13].

Antimicrobial Photodynamic Therapy (aPDT) is currently attracting attention and is being studied as a treatment for various microorganism infections [14,15,16,17]. The principle of PDT is based on the combined action of three non-toxic key elements, the so-called photosensitizer (PS), light with an adequate wavelength, and molecular oxygen. Upon selective irradiation at an appropriate wavelength with non-thermal light, PS generates indirect production of singlet oxygen/reactive oxygen species (ROS) responsible for killing bacteria [14,18]. aPDT has several advantages compared to classic antimicrobials and antibiotics [19,20]. Unlike antibiotics, which often target specific cellular components or metabolic pathways, aPDT operates through a non-specific mechanism. aPDT causes damage to multiple cellular components simultaneously, including proteins, lipids, and nucleic acids. This multi-pronged attack minimizes the chances of microorganisms developing resistance against all affected cellular structures. Moreover, in the case of antibiotics, resistance often arises due to mutations in specific genes or metabolic pathways. Since aPDT lacks such targeted pathways, there are no specific genes or mechanisms for microorganisms to evolve resistance against [14,21,22,23,24].

The intrinsic photochemical and physicochemical properties of PS are one of the key factors for the efficacy of aPDT. Among different organic dyes, cyanines (CYs) and squaraines (SQs), belonging to the class of polymethine dyes (PMD), have been proved to show excellent photophysical and photochemical properties including intense absorption and emission in organic solvent falling in the red and near-infrared region (NIR), matching the so-called phototherapeutic window, high extinction coefficients, and high fluorescence quantum yield. Moreover, they are usually cytocompatible in dark conditions and present low off-target effects and good photo- and thermal stability [25,26]. A limited number of papers on aPDT activities of squaraines series are reported: more than 20 years ago, Ramaiah et al. [27] described the promising phototoxicity of three halogenated SQ dyes in two strains of bacteria, *Salmonella typhimurium* TA100 and TA2638. More recently, the antibacterial activity of free and hydrogel-encapsulated asymmetric SQs on *Staphylococcus aureus* (*S. aureus*) and *Escherichia coli* (*E. coli*) bacteria were tested and recorded with inhibition greater than 90% and 80%, respectively [28], demonstrating a promising way for the application of squaraines for aPDT. This was further confirmed by the very recent paper of Delcamp’s group where an indolizine squaraine-based dye bearing sulfonate groups was studied for the detection and killing of drug-resistant bacteria by photothermal therapy (PTT) [29]. Another example was reported by the group of Kumar Pal [30,31], where they incorporated the indolenine-based dye inside ZIF-8 MOF and ZnO NPs to overcome the solubility issues and tendency to aggregate in physiological media of the squaraine.

To this purpose, our group proposed a different series of PMD [32,33] for application in anti-cancer PDT, among which a bromine-substituted indolenine-based squaraine (BrSQ) showed an excellent ROS production ability and excellent PDT activity against HT-1080 and MCF-7 tumor cell lines [32]. Based on these encouraging results, we decided to expand the applicability of this molecule and consider it as a potential candidate for aPDT. However, its tendency to self-aggregate in biological media can significantly affect its photochemical properties, thus limiting its application both in PDT and aPDT. Currently, the incorporation of hydrophobic molecules into organic and inorganic nanocarriers for drug delivery purposes is gaining growing attention by the scientific community [34]. In this regard, our group proposed the incorporation of squaraine dyes into a wide variety of nanosystems, such as mesoporous silica nanoparticles (MSNs) [35] and, more recently, Quatsomes nanovesicles [36].

In the view of increasing the biocompatibility of the nanosystems, we decided to investigate the use of poly lactic-co-glycolic acid (PLGA) NPs, widely recognized for their excellent safety profile in long clinical experience and excellent physicochemical properties [37,38,39] already exploited also in PDT [40,41,42,43].

**Figure 1:**
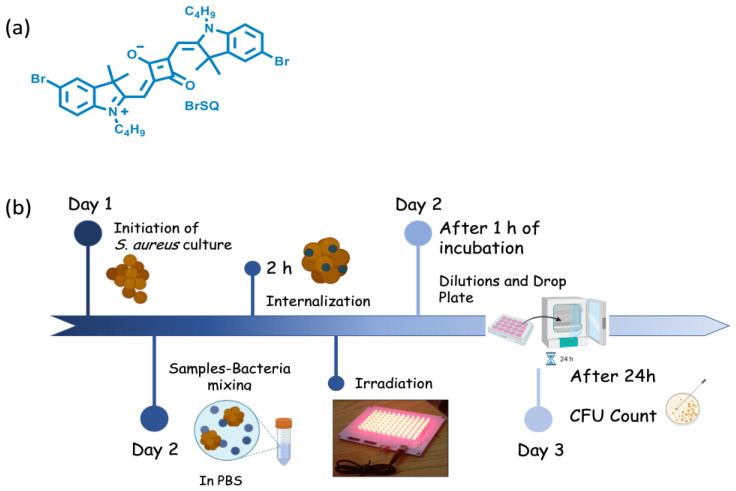
BrSQ structure (**a**); Schematic set-up of antibacterial PDT protocol (**b**).

Herein, BrSQ (represented in Figure 1a was incorporated into PLGA NPs to enhance its solubility in aqueous media and its aPDT activity was evaluated against representative Gram-positive *S. aureus* bacteria. We decided to apply a statistical multivariate design, such as the design of experiment (DoE), planning experiments with deliberate variables to identify the changes in the final results in order to optimize the preparation method of the BrSQ-loaded NPs. Two synthesis methods, i.e., single emulsion and nanoprecipitation, were optimized altering various formulation parameters, such as type of solvent, solvent ratio, concentration of PLGA, stabilizer and dye, and sonication power and time, and subsequently, we assessed the size, ζ-potential, yield, and encapsulation efficiency. Finally, the in vitro ROS production ability and aPDT activity of BrSQ-PLGA were evaluated. To the best of our knowledge, this is the first example of an SQ dye loaded into PLGA NPs for aPDT applications.

## 2. Materials and Methods

### 2.1. Chemicals and Reagents

Resomer^®^ RG 503 H (50:50) and Resomer^®^ RG 653 H (65:35), Poly (D, L-lactide-co-glycolide) with Mw 24,000–38,000, and Pluronic F-127 with Mw ~12,600 g/mol, were purchased from Merck (Merck KGaA, Darmstadt, Germany). Milli-Q water (resistivity 18.2 MΩcm) and all organic solvents were analytical grade. Bromine-substituted squaraine (BrSQ) was synthesized as already reported in the literature [32,44].

### 2.2. Design of Experiment

A design of experiment (DoE) approach based on multivariate analysis was performed with MODDE Pro 13 software (Sartorius AG, Gottingen, Germany) to investigate both the single emulsion and the nanoprecipitation methods for the preparation of BrSQ-loaded PLGA NPs. In particular, DoE was adopted to identify the most influencing experimental variables of the preparation procedure on selected interesting PLGA NPs features for drug delivery applications. Eight different independent variables (factors) were selected with their investigation ranges, as reported in Table 1.

The PLGA NPs features, defined as responses, were selected as (i) size of NPs; (ii) ζ potential of NPs surface; (iii) yield of NPs preparation; and (iv) dye entrapment efficiency (EE). Two similar D-optimal designs, each comprising 16 individual experimental trials and 3 replicates (center points), were adopted to investigate the preparation of SQ-loaded PLGA NPs through both nanoprecipitation (DoE-1) and single emulsion (DoE-2) methods. The designs differ exclusively based on the choice of organic solvents: acetone and acetonitrile are used in DoE-1, while dichloromethane and chloroform are employed in DoE-2. Multiple linear regression (MLR) was used for fitting the models to the experimental data [45]. Briefly, MLR adopts a numerical algorithm to compute the regression coefficients of each model by minimizing the squared difference between the response values and the linear prediction model.

The goodness of the model fit was assessed using the coefficient of determination (R^2^), which denotes the fraction of the response that is explained by the model [46].

### 2.3. Synthesis of BrSQ-Loaded PLGA Nanoparticles

The protocol used to produce empty and BrSQ-PLGA nanoparticles is based on modified nanoprecipitation (DoE-1) and modified single emulsion (DoE-2) with sonication probe (Sonics Vibra Cell VC375 Ultrasonic Processor, Sonics & Materials, inc., Newtown, CT, USA)) [47,48]. Briefly, in both cases, the organic phase is prepared by dissolving Resomer^®^ (65:35) or Resomer^®^ (50:50) in acetone or acetonitrile for DoE-1 and DCM or chloroform for DoE-2, according to the amount reported in Table 2 and Table 3. The aqueous phase is prepared for both DoEs solubilizing Pluronic F-127 in water (10 mL), according to the total amount specified in Table 2 and Table 3. Then, the organic phase is poured into the aqueous one and instantly the sonication is performed, maintaining the beaker on ice at pulse mode with a 2 s on and 2 s off cycle, varying the output power and total sonication time following the DoEs. The same protocol was followed for the preparation of dye-loaded PLGA except for the dissolution of the BrSQ, at a given amount, in the organic phase prior to the addition to the aqueous phase. During the sonication, it is imperative to cool down the container with ice to avoid an increase in temperature, and thus the evaporation of the organic solvent, and cover the vial with aluminum foil to protect the dye from the light. Then, the organic solvent is removed under gentle stirring at room temperature in the fume hood for 4–6 h. Finally, the purification of the nanosuspension was performed by centrifugation (JOUAN MR23i Benchtop High Speed Centrifuge Thermo Scientific, Waltham, MA, USA) at 10,000 rpm for 30 min, washed with deionized water three times, and re-suspended in water before freeze-drying.

#### Yield and Encapsulation Efficiency (EE%)

The amount of dye in PLGA NPs was calculated by a direct method using UV-Vis spectroscopy. A calibration curve of free BrSQ in DMSO (at the absorption wavelength of 652 nm) was prepared. Then, 2 mg of lyophilized BrSQ-PLGA NPs were dissolved in 30 mL of DMSO and sonicated by using an ultrasonic bath (Ultrasonic Cleaning Unit, output 45 kHz, VWR, Milan, Italy) for 5 min to force the breaking of the NPs and the release of the dye in the solution. By using the Lamber–Beer equation (molar extinction coefficient, ε, of BrSQ = 441,667.5 M^−1^cm^−1^), the concentration of the released dye was calculated. The final entrapment efficiency and drug loading capacity were calculated using standard Equations (1) and (2) [49]:

(1) $$\mathrm{Encapsulation}\;\mathrm{efficacy}\;\mathrm{EE}\;(\%)=\frac{mass\;of\;BrSQ\;(encapsulated)}{mass\;of\;BrSQ\;initially\;used\;}\;\cdot 100$$

(2) $$\mathrm{Drug}\;\mathrm{loading}\;\mathrm{Capacity}\;\mathrm{LC}\;(\%)=\;\frac{mass\;of\;BrSQ\;(encapsulated)}{mass\;of\;BrSQ\;\;loaded\;PLGA\;}\cdot 100$$

To calculate the NPs yield, the amount of the obtained NPs, after freeze drying, was weighted, and compared to the initial amount of polymers and BrSQ, by following Equation (3):

(3) $$\mathrm{Nanoparticle}\;\mathrm{yields}\;(\%)=\frac{Weight\;of\;freeze\text{d−d}ried\;NPs}{Initial\;weight\;of\;polymer\;and\;BrSQ}\;\cdot 100$$

### 2.4. Characterization of BrSQ-Loaded PLGA Nanoparticles

#### 2.4.1. Nanoparticle Tracking Analyzer (NTA)

The mean particle diameter (MPD), polydispersity (PDI), and zeta potential (ζ-potential) of empty and loaded NPs were analyzed through a nanoparticle tracking analyzer (ZetaView^®^ PMX-120 mono-laser, YG-488, Malvern Panalytical, Malvern, UK) equipped with software version 8.05.14_SP7. Measurements were carried out using a laser beam (λ 488 nm) at 25 °C and the following set-up: mobility profile measurement, 11 positions, Max Area, 10,000, Min Area, 10, Min Brightness, 25. Samples were injected into the sample port (1 mL) after calibration with polystyrene (1 mL of 100 nm size). The ζ-potential of the NPs was measured at the 2 stationary layers.

#### 2.4.2. Field Emission Electron Microscopy (FE-SEM)

The size and shape of both empty and dye-loaded NPs were observed with field emission electron microscopy (FE-SEM TESCAN S9000G, Brno, Czech Republic), Schottky Emitter, Resolution “in-beam SE mode”: 0.7 nm at 15 keV. The dry powder was directly dispersed on a conductive carbon tape and coated with a Cr layer (7 nm).

#### 2.4.3. UV-Vis Spectroscopy and Determination of the Molar Extinction Coefficient

UV-Vis spectra were recorded at room temperature on a Shimadzu UV-1700 PharmaSpec (Shimadzu Europa GmbH, Duisburg, Germany) in the range of 500–800 nm using quartz cuvettes with a 1 cm pathway length. BrSQ or BrSQ-PLGA were dissolved in water in order to investigate their solubility. Moreover, in order to evaluate the molar extinction coefficient, a stock solution of BrSQ (0.5 mM) in DMSO was prepared and then different diluted solutions were obtained by taking proper aliquots of the stock solution. The diluted solutions (0.5, 1.0, 1.3, 1.7, 2.0, 3.0 μM) were measured by UV-Vis spectroscopy. The absorbance intensities of each solution at the λ_max_ were plotted versus the sample concentration. A linear fit was applied to determine the molar extinction coefficient (ε) as the slope of the line. The analysis was performed in duplicate. The obtained data were considered acceptable when the difference between the measured log ε was less or equal to 0.02 in respect to their average.

#### 2.4.4. Measurement of Reactive Oxygen Species (ROS)

A 1,3-diphenylisobenzofuran (DPBF) probe was used to preliminarily evaluate the ability of the BrSQ before and after the incorporation into PLGA NPs to produce ROS, following the protocol reported in the literature [33]. Stock solutions of DPBF were prepared in DMSO as well as the stock solution of the free BrSQ. On the other hand, the stock solution of BrSQ-PLGA can be directly prepared in phosphate buffer saline (PBS, 2 mM, pH 7.4). Proper dilutions were then performed in PBS directly in a quartz cuvette with a 1 cm light path (final concentration of DPBF: 25 μM; BrSQ: 5 μM). The cuvette with solutions was covered and irradiated in an aerated solar box (Solarbox 3000e, 250 W xenon lamp, CO.FO.ME.GRA, Milan, Italy) with a 250 W lamp. In order to avoid the DPBF degradation and allow just the BrSQ excitation, light was filtered using an optical filter (515 nm cut-off). At different time points, absorption spectra were recorded using a Cary 300 Bio spectrophotometer instrument (Varian, Santa Clara, CA, USA). The decrease in the DBPF absorption contribution at 415 nm was plotted as a function of irradiation time.

### 2.5. Antimicrobial Assays

*Staphylococcus aureus* (*S. aureus*, ATC 29,213 laboratory strain) was selected as the representative Gram-positive bacteria model to evaluate the antimicrobial properties of the BrSQ and BrSQ-PLGA. The experimental procedure is schematically illustrated in Figure 1b.

The initiation of the bacterial culture begins with the extraction of one colony-forming unit (CFU) of *S. aureus* and its culture overnight in 20 mL Todd Hewitt Broth (THB) culture medium at 37 °C and 100 rpm. The *S. aureus* working concentration (2 × 10^6^ bacteria/mL) was assessed by measuring the inoculum turbidity by optical density at 600 nm, OD600, with a visible spectrophotometer (Photoanalyzer D-105, Dinko instruments, Barcelona, Spain). For this, it was necessary to first dilute the inoculum with THB (1:6), culture it for 15 min, evaluate its turbidity, and finally dilute it (1:600) with PBS 1 to obtain a working concentration of 2 × 10^6^ bacteria/mL.

The stock solutions of 1.5 mM (1 mg/mL) of BrSQ in DMSO and 1 mg/mL of PLGA and BrSQ-PLGA in PBS1× (pH 7.4 and pH 5.5) were prepared. Afterwards, 100 µL of the prepared inoculums were replenished with 100 µL of varied concentrations of BrSQ, BrSQ-PLGA, and empty PLGA NPs (100 nM, 500 nM, 1000 nM, 2 µM, 3 µM, and 5 µM) in a P96-well plate and incubated at 37 °C for 2 h to allow the dye internalization. The amount of the DMSO in the dye solutions added to the bacteria suspensions did not exceed 1%. The irradiation was then performed using an illumination system consisting of a RED LED array (light source with excitation wavelength at 640 nm) composed of 96 LEDs arranged in 12 columns and 8 rows. Cicci Research s.r.l. (Grosseto, Italy) designed and manufactured this compact LED array-based system specifically for this kind of test. Fluence 6.2 J/cm^2^, irradiance 7 mW/cm^2^, time 15 min, and incubation time 1 h at 37 °C were the parameters used for standard treatments. Some treatments were also performed at higher power, increasing the fluence and irradiance at 15.7 J/cm^2^ and at 17.4 mW/cm^2^, respectively. After incubation for 1 h at 37 °C (after irradiation for 15 min at 640 nm), serial dilutions of aliquots of the treated samples were prepared and 10 μL of the final solution of each condition were seeded on Tryptic Soy Agar (TSA) plates. After 24 h of culture at 37 °C and under static conditions, the CFUs were counted. To assess the potential toxicity under dark conditions, the same procedure was performed without 640 nm irradiation. Finally, three independent experiments were carried out for each experimental condition.

#### Statistical Analysis

Data were reported as an average of 3 independent experiments with the corresponding standard deviations (SD). Statistical analyses were performed using the two-tailed Student’s *t*-test (Excel, Microsoft Office Professional Plus 2016) to determine the difference between the two groups (dark vs. light). Statistical significance values were * *p* < 0.05 (significant), ** *p* < 0.01 (moderately significant), *** *p* < 0.005 (highly significant), and **** *p* < 0.001 (very highly significant).

## 3. Results and Discussion

In the present work, empty and SQ-loaded PLGA NPs were prepared and investigated as potential PS for antimicrobial PDT. As a proof of concept, we chose a well-known and active SQ dye, i.e., BrSQ, which already showed a high ROS production in some previous works [32,36]. Despite its efficacy, this PS presents some solubility and aggregation issues in 100% aqueous media needing the use of DMSO for its complete solubilization. To overcome these issues, previous attempts were performed, incorporating BrSQ into MSNs [35]. For the present study, we chose PLGA NPs due to their biodegradability and biocompatibility, as well as their Food and Drug Administration (FDA) and European Medicine Agency (EMA) approval in drug delivery systems for parenteral administration. Several methods of preparation of empty and loaded PLGA NPs have been reported in the literature [37,38,43,48], among which single emulsions and nanoprecipitation were suggested as the best formulation methods for encapsulating hydrophobic PSs [47]. The two methods have been optimized with a statistical multivariate design, such as the DoE, able to provide the maximum information while requiring a minimum number of experiments.

### 3.1. Experimental Design

**Figure 2:**
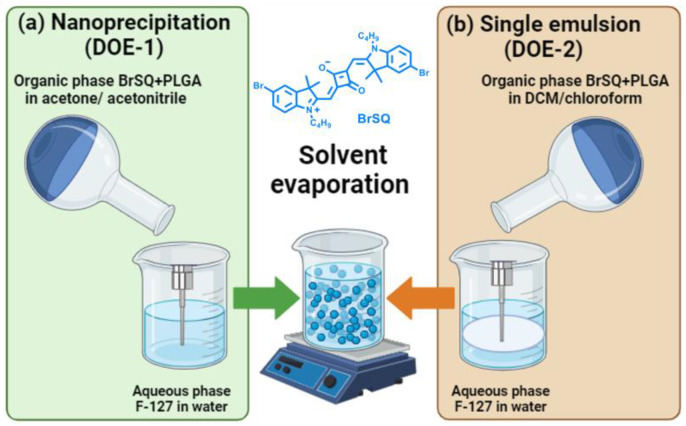
Synthetic workflow for the BrSQ-loaded PLGA nanoparticles preparation via the (**a**) nanoprecipitation (DoE-1) technique and (**b**) single emulsion method (DoE-2).

Finding the optimal combination of different parameters for a specific drug and formulation involves conducting experiments to explore these parameters systematically and singularly. It is essential to strike a balance that maximizes entrapment efficiency while avoiding issues like drug degradation, excessive particle size reduction, or instability in the formulation. To this purpose, aiming to study the experimental variables that most influence the synthesis of SQ-loaded PLGA nanoparticles via both nanoprecipitation and single emulsion methods, a multivariate statistical approach based on the design of experiment (DoE) was adopted (Figure 2) [50,51].

According to literature data and a few preliminary trials (not reported here), eight experimental variables were selected from the synthetic procedure of the SQ-loaded PLGA nanoparticles and their effects on the synthesized NPs were evaluated by means of DoE. The selected variables (factors) are both quantitative and qualitative and are listed together with their range of investigation in Table 1. Two similar DoEs have been performed to investigate the SQ-loaded PLGA NPs preparation via both nanoprecipitation (DoE-1) and single emulsion (DoE-2) methods. The two designs differ only in the solvent type selection due to the different procedure adopted in the PLGA NPs synthesis. The organic solvents were chosen among those commonly reported in the literature with these kinds of synthetic methods: acetone and acetonitrile for the nanoprecipitation method in DoE-1, while dichloromethane and chloroform for the single emulsion method in DoE-2. All these solvents have been usually proposed in the literature for PLGA NPs synthesis [37,52].

Concerning the other variables, different amounts of two PLGA copolymers with different lactide/glycolide ratios (L/G) of 65:35 and 50:50 have been selected. The different ratios could influence the drug release rate, since higher lactide-based PLGA polymers have a more hydrophobic nature, leading to a denser and more slowly degrading matrix and thus resulting in longer drug release profiles and potential extended therapeutic effects. Moreover, even if generally considered biocompatible, the L/G ratio can affect the biocompatibility and toxicity of PLGA NPs [37,52]. The stabilizer, Pluronic F-127, was chosen after a few preliminary trials (not reported here) due to its tendency to form particles with smaller mono-distributed size compared to polyvinyl alcohol (PVA) [53,54]. Furthermore, the literature confirmed that Pluronic F-127, commonly employed in the preparation of PLGA NPs, helps to create a stable oil-in-water emulsion, preventing the rapid coalescence of PLGA droplets and increasing the NPs size uniformity. Moreover, PLGA NPs prepared with Pluronic F-127 demonstrated a higher therapeutic effect in overcoming multidrug resistance (MDR) than NPs formulated using PVA [54,55]. For these reasons and in order not to further increase the complexity of the DoE investigation, the stabilizer type was not included as a variable, and Pluronic F-127 was used for all the experiments. Only the quantity of the stabilizer (0.5–1.5% wt) used in the synthesis was investigated due to its proven role in NPs formation to control the entrapment efficiency and particle size, prevent aggregation, and enhance stability [53].

Usually, the study of such a large number of variables requires a consequent large number of experiments. However, the advantage of DoE is to require a limited number of trials, and in this study a D-optimal design was selected for both DoE-1 and DoE-2, which required only 16 experimental trials. An additional three trials, the so-called center points, were performed in the center of the experimental domain (quantitative variables set at 0 level, qualitative variables arbitrarily selected) to evaluate the reproducibility and the experimental error of the procedure. The lists of experimental trials performed with the nanoprecipitation (DoE-1) and single emulsion (DoE-2) methods are reported in Table 2 and Table 3, respectively.

The effect of the variables on the PLGA NPs synthesis was evaluated by measuring four different responses: (i) size of NPs; (ii) ζ-potential of NPs surface; (iii) yield of NPs synthesis; and (iv) dye encapsulation efficiency.

The particle size, ζ-potential, the yield of NPs, and the dye encapsulation efficiency (EE) were measured and calculated as reported in Section 2.3.

#### 3.1.1. Nanoprecipitation Method (DoE-1)

The DoE analysis was performed to identify the influential variables on the BrSQ-PLGA NPs synthesis based on the nanoprecipitation method. All the experimental conditions of the performed trials are reported in Table 2, together with the corresponding response values obtained and considered for the DoE analysis.

**Table 2:** Synthetic conditions used in DoE-1 and corresponding results of the BrSQ-PLGA NPs synthesis via nanoprecipitation method. Ac: acetone, ACN: acetonitrile. “Qty” means quantity.

Exp. N°	PLGA Qty (mg)	PLGA Type	Stabilizer Qty (wt%)	O/A Ratio	Dye Qty (mg)	Solvent Type	Sonic Time (min)	Sonic Power (W)	Size (Nm)/N° of Part	ζ-Pot (mv)	Yield (%)	EE (%)
1	30	65:35	0.5	0.3	0.5	Ac	0.5	120	131/96	−20	62	1.5
2	70	65:35	0.5	0.3	0.5	ACN	3.5	240	155/73	−15	50	1.4
3	30	50:50	0.5	0.3	1.5	Ac	3.5	240	123/159	−29	72	0.1
4	70	50:50	0.5	0.3	1.5	ACN	0.5	120	162/181	−27	38	1
5	30	65:35	1.5	0.3	1.5	ACN	3.5	120	138/92	−33	14	0.3
6	70	65:35	1.5	0.3	1.5	Ac	0.5	240	143/71	−26	50	0.1
7	30	50:50	1.5	0.3	0.5	ACN	0.5	240	147/107	−36	34	3.8
8	70	50:50	1.5	0.3	0.5	Ac	3.5	120	149/95	−34	39	8.2
9	30	65:35	0.5	0.7	1.5	ACN	0.5	240	133/97	−38	57	1.7
10	70	65:35	0.5	0.7	1.5	Ac	3.5	120	127/115	−25	72	1.5
11	30	50:50	0.5	0.7	0.5	ACN	3.5	120	142/60	−36	35	23.6
12	70	50:50	0.5	0.7	0.5	Ac	0.5	240	115/64	−23	71	0.7
13	30	65:35	1.5	0.7	0.5	Ac	3.5	240	114/136	−35	32	0.5
14	70	65:35	1.5	0.7	0.5	ACN	0.5	120	125/96	−16	54	0.3
15	30	50:50	1.5	0.7	1.5	Ac	0.5	120	111/121	−23	46	9.8
16	70	50:50	1.5	0.7	1.5	ACN	3.5	240	146/113	−34	28	0.5
17	50	50:50	1	0.5	1	Ac	2	180	119/107	−40	37	6
18	50	50:50	1	0.5	1	Ac	2	180	119/110	−36	51	0.2
19	50	50:50	1	0.5	1	Ac	2	180	122/102	−33	36	3.8

From a preliminary observation of the experimental results, the variations in size and ζ-potential of the synthesized BrSQ-loaded PLGA NPs are quite low, ranging from 111 nm to 162 nm and from −15 mV to −40 mV, respectively, over the whole experimental domain. In all cases, the obtained size and ζ-potential values are considered suitable for biomedical application; in fact, as a drug delivery system, NPs in the range 20–200 nm are often preferred to enhance tumor targeting and avoid rapid clearance by the immune system [56]. Moreover, the obtained ζ-potential values ensure electrostatic repulsion between particles allowing to maintain colloidal stability and prevent particle aggregation [57,58]. Differently, the yield values of NPs synthesis show higher variation ranging from 7% to 72%. Concerning the entrapment efficiency of BrSQ dye into the PLGA NPs, all values are generally low (<10%) with a maximum of 23.6%.

**Figure 3:**
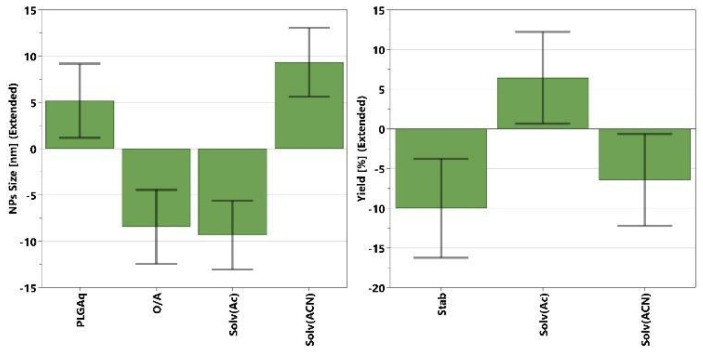
Significant coefficients (green bars) and corresponding confidence interval (black lines) for the NPs size (**left**) and yield (**right**) models computed for DoE-1 by MODDE software.

The multivariate analyses on the nanoprecipitation method show generally poor data fitting and low significance (low R^2^ values ≤ 0.8) for all responses, indicating the computed models are able to describe only a small fraction of the observed variation in the experimental data. Only a few factors showed a significant effect on the NPs size and yield responses, while no significant effects were observed on the ζ-potential and EE. The significant coefficients for NPs size and yield models are reported in Figure 3. Coefficients’ values are represented by green bars with error bars at a 95% confidence interval. They show whether a change in the factor value (from middle to higher value) causes a significant variation (positive or negative) on the selected responses. A higher value of coefficient (green bar) corresponds to a higher variation of response, and therefore a greater effect on it. A positive value means that the response (i.e., EE%, size, or yield) will increase by increasing that factor, while negative values represent the opposite, meaning that increasing that value, the response will decrease. For the NPs size (Figure 3, left), the most influential factor is the solvent type, and syntheses performed with acetonitrile tend to yield larger particles than those performed with acetone. The NPs size tends to increase not only with a decrease in the O/A ratio but also with an increase in the amount of PLGA, although the latter has a less pronounced influence.

Concerning the yield of NPs synthesis (Figure 3, right), only two significant factors can be identified: the solvent type and the stabilizer quantity. In particular, the yield is improved by using acetone as the organic phase, and a low amount of stabilizer. Even though the DoE analysis gives some tips in modifying the NPs size and improving the synthesis yield, the entrapment efficiencies obtained with the nanoprecipitation method were generally low and not clearly dependent from the experimental variables considered in DoE investigation.

The most probable explanation for all the aforementioned observations could be related to the low solubility of BrSQ dye in the organic phase, i.e., acetone and acetonitrile. A lower amount of dye dissolved in solution causes a subsequent lower and uneven loading of dye into the PLGA NPs. For this reason, the nanoprecipitation method with acetone and acetonitrile appears not suitable for the synthesis of BrSQ-loaded PLGA NPs and this synthetic approach was not further investigated.

#### 3.1.2. Single Emulsion Method (DoE-2)

Similarly to the evaluation performed with the nanoprecipitation method, a second DoE analysis was conducted on BrSQ-PLGA NPs synthesis based on a single emulsion method. All the experimental conditions of the performed trials are reported in Table 3, together with the corresponding response values obtained and considered for the DoE analysis.

**Table 3:** Synthetic conditions used in DoE-2 and corresponding results of the BrSQ-PLGA NPs synthesis via single emulsion method. DCM: dichloromethane, CLF: chloroform. “Qty” means quantity.

Exp. N°	PLGA Qty (mg)	PLGA Type	Stabilizer Qty (wt%)	O/A Ratio	Dye Qty (mg)	Solvent Type	Sonic Time (min)	Sonic Power (W)	Size (nm)/N° of Part	ζ-Pot (mv)	Yield (%)	EE (%)
1	30	65:35	0.5	0.3	0.5	DCM	0.5	120	160/103	−30	28	70
2	70	65:35	0.5	0.3	0.5	CLF	3.5	240	145/58	−31	12	37
3	30	50:50	0.5	0.3	1.5	DCM	3.5	240	157/175	−26	35	11
4	70	50:50	0.5	0.3	1.5	CLF	0.5	120	139/159	−10	6	15
5	30	65:35	1.5	0.3	1.5	CLF	3.5	120	160/139	−35	34	25
6	70	65:35	1.5	0.3	1.5	DCM	0.5	240	155/112	−32	72	76
7	30	50:50	1.5	0.3	0.5	CLF	0.5	240	177/120	−27	65	33
8	70	50:50	1.5	0.3	0.5	DCM	3.5	120	157/112	−34	28	58
9	30	65:35	0.5	0.7	1.5	CLF	0.5	240	153/65	−27	33	52
10	70	65:35	0.5	0.7	1.5	DCM	3.5	120	167/97	−26	12	56
11	30	50:50	0.5	0.7	0.5	CLF	3.5	120	162/78	−30	7	4
12	70	50:50	0.5	0.7	0.5	DCM	0.5	240	141/86	−20	26	49
13	30	65:35	1.5	0.7	0.5	DCM	3.5	240	137/65	−19	24	28
14	70	65:35	1.5	0.7	0.5	CLF	0.5	120	159/104	−28	21	68
15	30	50:50	1.5	0.7	1.5	DCM	0.5	120	187/126	−25	85	65
16	70	50:50	1.5	0.7	1.5	CLF	3.5	240	151/152	−30	8	48
17	50	50:50	1	0.5	1	DCM	2	180	170/122	−31	43	36
18	50	50:50	1	0.5	1	DCM	2	180	169/120	−32	45	39
19	50	50:50	1	0.5	1	DCM	2	180	171/127	−20	39	31

As previously observed in DoE-1, the size and the ζ-potential of NPs synthesized via single emulsion show low variability over the whole experimental domain. The NPs size ranges from 137–187 nm, generally yielding slightly higher values than nanoprecipitation, while the ζ-potentials range similarly to DoE-1, from −10 mV to −35 mV. Thus, even in this case, all measured values for size and ζ-potential are considered suitable for the design of a drug delivery system. Conversely, the measured yields and EEs exhibit greater variation, with values ranging from 6% to 85% and from 4% to 76%, respectively. Moreover, the maximum drug-loading capacity (LC) recorded and used was 2.2%.

**Figure 4:**
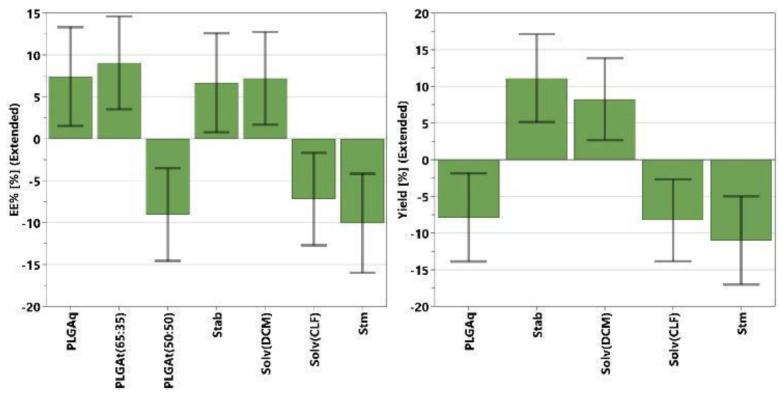
Significant coefficients (green bars) and corresponding confidence intervals (black lines) for the EE (**left**) and yield (**right**) models, computed by MODDE software, for the single emulsion method (DoE-2).

To deepen the study on the single emulsion method, DoE analysis was performed on the experimental data, similarly to DoE-1. The analyses on the size and ζ-potential of PLGA NPs show very poor data fitting (R^2^ < 0.4), indicating that no significant factors were identified to influence the two responses. On the contrary, the analyses on the yield and EE show satisfactory data fitting with R^2^ around 0.7, and several factors have been observed to be influential on the final responses. The significant coefficients of the EE model, in order of magnitude, are sonication time > PLGA type > PLGA quantity > solvent type > stabilizer (Figure 4, left). In terms of quantitative factors, sonication time shows a negative effect on the entrapment efficiency, while increasing the amount of PLGA and stabilizer positively affects the EE.

The dye amount, O/A ratio, and sonication power, within the studied ranges, do not show significant effects on the EE, and their coefficients have been removed from the EE model. Regarding the qualitative factors, PLGA type, and solvent, NPs synthesized with PLGA 65:35 using dichloromethane generally exhibit a higher EE compared to those with PLGA 50:50 and chloroform.

Differently for the yield, the significant coefficients, in order of magnitude, are stabilizer > sonication time > solvent type > PLGA quantity (Figure 4, right). A high amount of stabilizer and low PLGA quantity with dichloromethane as the organic solvent and a short sonication time allow for obtaining higher synthetic yields. Based on the DoE-2 results, experiment number 6 (PLGA amount: 70 mg; PLGA type: 65:35; stabilizer amount: 1.5 wt%; O/A ratio: 0.3; dye amount: 1.5 mg; solvent type: DCM; sonication time: 0.5 min; sonication power: 240 W) showed the best compromise in terms of EE% and yield; thus, it has been selected for the preparation of BrSQ-PLGA NPs that have been further characterized by FE-SEM and spectroscopic techniques and tested to evaluate their aPDT activity.

### 3.2. Physicochemical Characterization of BrSQ-Loaded PLGA NPs

**Figure 5:**
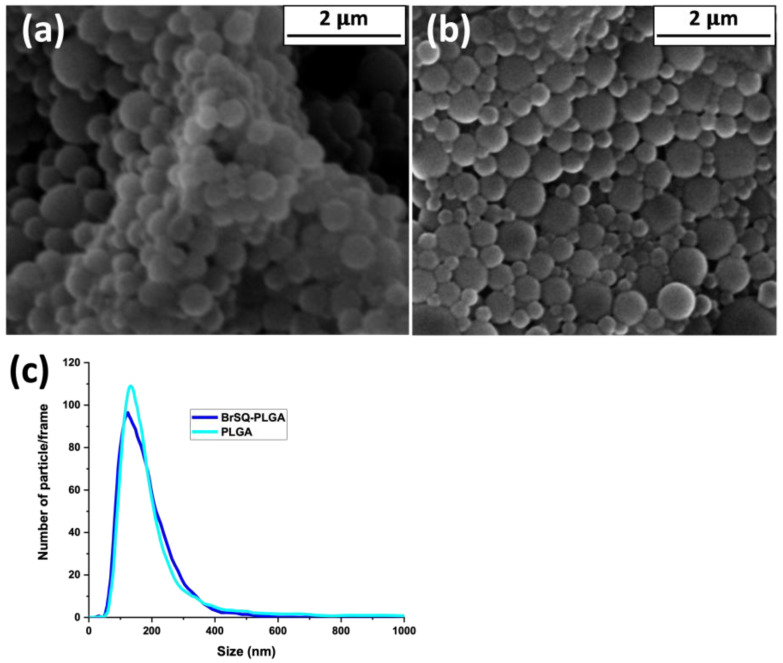
FE-SEM images of (**a**) PLGA, (**b**) BrSQ-PLGA, and (**c**) nano-tracking analysis of PLGA and BrSQ-PLGA.

A morphological observation of empty and BrSQ-PLGA NPs was performed using FE-SEM (Figure 5). As shown in Figure 5a, empty PLGA NPs show a spherical morphology with a size around 200 nm. The presence of BrSQ does not alter the spherical shape nor the size (Figure 5b). The FE-SEM observation confirmed the size and good distribution of both empty and loaded NPs obtained by NTA (Figure 5c).

### 3.3. Spectroscopic Characterization of BrSQ-Loaded PLGA NPs

#### 3.3.1. Water Solubility Analysis

**Figure 6:**
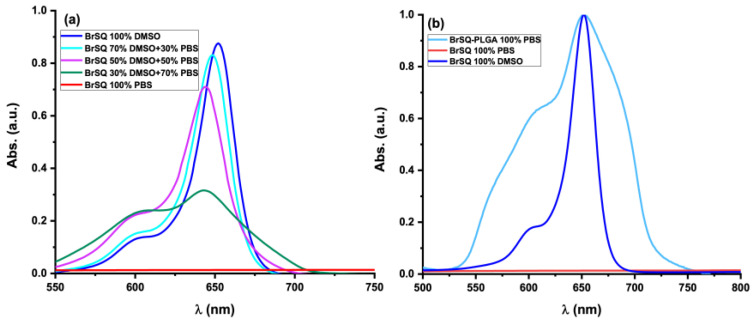
(**a**) UV-Vis spectra of BrSQ dissolved in different percentages of DMSO and PBS, (**b**) comparison of the UV-Vis spectra of free BrSQ in 100% DMSO, 100% PBS, and BrSQ-PLGA NPs in 100% PBS. The concentration of BrSQ was kept constant for all the measurements.

The spectroscopic absorption of free dye BrSQ and dye-loaded PLGA NPs were evaluated. The absorption spectra of both free dye BrSQ and dye-loaded PLGA NPs were assessed. In Figure 6a, the absorption spectrum of BrSQ in DMSO reveals a prominent absorption peak characteristic of squaraine dyes, with an absorption maximum centered at 652 nm. However, with an increasing PBS percentage in the solution, the absorption peak gradually decreases showing a simultaneous hypsochromic shift. Moreover, the absorption intensity of the hypsochromic shoulder increases, indicating the formation of H aggregates. After 50% PBS, the squaraine-like profile of the absorption spectrum further decreases, up to a complete disappearance of the typical spectroscopic properties observed in organic solvents when the amount of PBS is 100%, due to the impossibility to dissolve the BrSQ. Conversely, after the incorporation of the dye within PLGA NPs, its solubility in aqueous media improves, as depicted in Figure 6b. The slight alterations on the absorption spectrum compared to free BrSQ in DMSO can be ascribed to particle scattering in the suspension.

#### 3.3.2. ROS Measurement Analysis In Vitro

**Figure 7:**
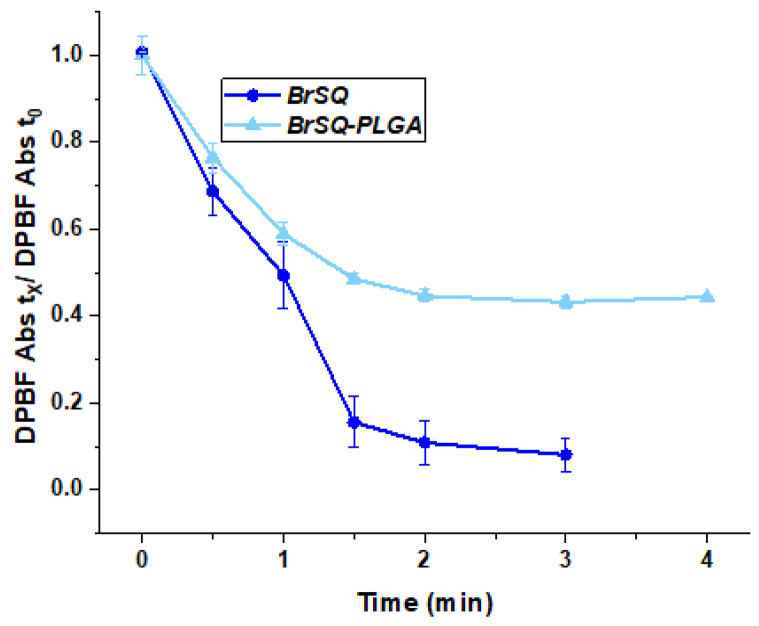
Decay of the absorption band of DPBF at 418 nm as a function of the irradiation time in the presence of free BrSQ and BrSQ-loaded PLGA NPs. Data are reported as an average of 3 independent experiments and error bars represent the standard deviation.

The evaluation of the ability of the dye-loaded PLGA NPs to generate ROS was performed by using 1,3-diphenylisobenzofuran (DPBF) as a probe and compared to the ROS production of free BrSQ already reported in the literature [32]. As shown in Figure 7, the BrSQ-loaded PLGA NPs maintain a good ability to quickly generate ROS compared to the free dye. However, the presence of the polymer matrix scatters the light, decreasing the light dose able to excite the SQ, which could explain the difference in ROS production. It is worth noting that the evaluation of the ROS production is performed in 100% PBS in the case of the BrSQ-PLGA NPs while the free dye needs to be dissolved in 100% DMSO and subsequently diluted in PBS to get a final DMSO concentration lower than 1%. However, if the ROS production of the free dye was compared with that of BrSQ-PLGA in 100% PBS, the ROS production would be significantly higher for the BrSQ-PLGA system. In fact, the ROS production of the free dye in 100% PBS would not be possible due to the complete insolubility of the dye in PBS and would therefore be nil. The incorporation of the dye into the PLGA NPs allows for the evaluation of ROS in a condition similar to the physiological ones, which would not be possible with free dye. This confirms the utility and necessity of incorporating the dye for potential biological applications.

### 3.4. In Vitro Antimicrobial PDT

**Figure 8:**
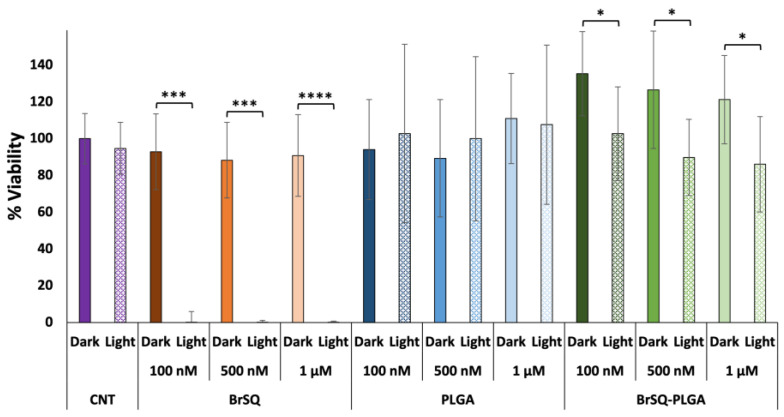
In vitro antimicrobial effect of different concentrations of BrSQ, PLGA, and BrSQ-PLGA on *S. aureus* in the dark and after irradiation (640 nm LED, fluence 6.2 J/cm^2^, irradiance 7 mW/cm^2^ for 15 min). For dye-loaded PLGA NPs, the concentrations refer to dyes incorporated into PLGA (from 100 nM to 1 µM) in PBS buffer, pH 7.4. Data are reported as an average of 3 independent experiments. *p* values: * *p* < 0.05, ** *p* < 0.01, *** *p* < 0.005, and **** *p* < 0.001.

The photodynamic effect of free BrSQ and BrSQ-PLGA NPs against Gram-positive *S. aureus* bacteria was investigated under different conditions (i.e., dye concentration, irradiation time, irradiation power, and pH of the medium). As a first trial, based on previous works performed on cancer cell lines [32,33,36], the PDT treatment was set up with the following parameters: concentration ranging from 100 nM to 1 µM in PBS buffer pH 7.4, irradiation time 15 min, irradiation power at 640 nm with a fluence of 6.2 J/cm^2^, and irradiance 7 mW/cm^2^. As reported in Figure 8, the free dye shows no significant toxicity in dark conditions and a strong bacteria inactivation after irradiation at all tested concentrations. As expected, since PLGA NPs are biocompatible and FDA-approved nanosystems, empty PLGA NPs did not show any toxicity neither in the dark nor upon irradiation. Surprisingly, contrary to what was observed in the ROS production experiments, BrSQ-loaded PLGA NPs showed no significant effect on the bacteria viability after irradiation in such conditions.

**Figure 9:**
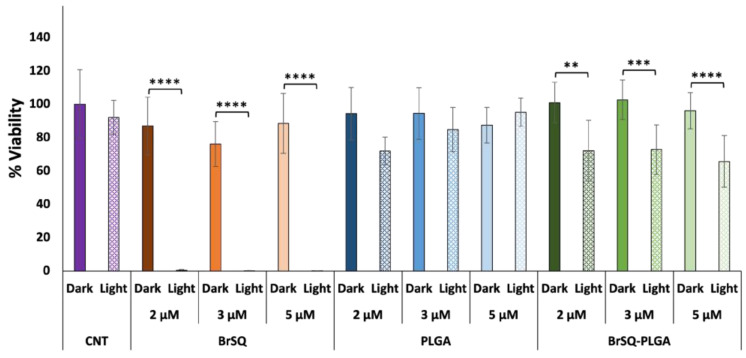
In vitro antimicrobial effect of different concentrations of BrSQ, PLGA, and BrSQ-PLGA on *S. aureus* in the dark and after irradiation (640 nm LED, fluence 6.2 J/cm^2^, irradiance 7 mW/cm^2^ for 15 min). For dye-loaded PLGA NPs, the concentrations refer to dyes incorporated into PLGA (from 2 µM to 5 µM) in PBS buffer, pH 7.4. Data are reported as an average of 3 independent experiments. *p* values: * *p* < 0.05, ** *p* < 0.01, *** *p* < 0.005, and **** *p* < 0.001.

With the aim of increasing the aPDT activity for loaded PLGA NPs, a second trial was performed by increasing the dye-loaded NPs concentration to test a final dye concentration of 2, 3, and 5 µM (Figure 9). While the free dye still showed strong toxicity after aPDT irradiation, unfortunately the BrSQ-loaded PLGA NPs did not show hardly any bacterial inactivation. This effect could be probably ascribed to the light scattering originating from the polymer shell that absorbs part of the light, thus limiting the excitation of the dye and the following ROS production, as already observed in Figure 7.

**Figure 10:**
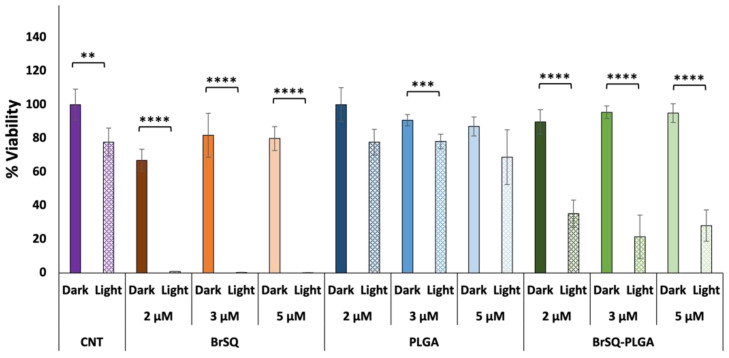
In vitro antimicrobial effect of different concentrations of BrSQ, PLGA, and BrSQ-PLGA on *S. aureus* in the dark and after irradiation (640 nm LED, fluence 15.7 J/cm^2^, irradiance 17.4 mW/cm^2^ for 15 min). For dye-loaded PLGA NPs, the concentrations refer to dyes incorporated into PLGA (from 2 µM to 5 µM) in PBS buffer, pH 7.4. Data are reported as an average of 3 independent experiments. *p* values: * *p* < 0.05, ** *p* < 0.01, *** *p* < 0.005, and **** *p* < 0.001.

To overcome these issues, since the role of the light dose seems to contribute to the bacterial viability [59,60], the irradiance was increased up to 17.4 mW/cm^2^ (fluence 15.7 J/cm^2^) by maintaining the concentration of 2, 3, and 5 µM. As reported in Figure 10, in this case the viability percentage of bacteria after irradiation notably decreased up to 60–80% compared to the control, highlighting that a high dose of light can be effective for the treatment.

Since it is known that during a bacterial infection the pH can decrease around 5.5–6 [61], to exploit this environment and boost the aPDT effect, we decided to explore the effect of the acidic pH, keeping the previous setup (i.e., high irradiance and fluence) in order to perform the experiments in a more realistic scenario. Moreover, PLGA NPs are known to degrade via hydrolysis of ester linkages [62] and, along with higher temperatures and longer time exposure, this process can be further accelerated when exposed to acidic conditions [63]. We supposed that using an acidic pH could further help in reducing the bacterial count, as the polymer matrix may become more porous due to swelling phenomena in an acidic aqueous environment, thereby allowing greater light penetration and, thus, the dye excitation.

**Figure 11:**
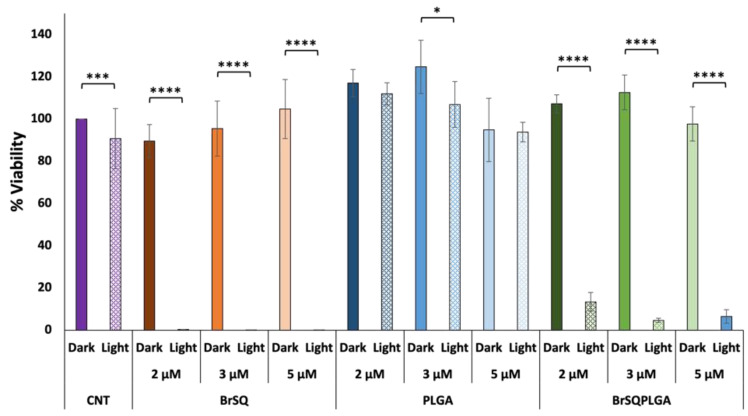
In vitro antimicrobial effect of different concentrations of BrSQ, PLGA, and BrSQ-PLGA on *S. aureus* in the dark and after irradiation (640 nm LED, fluence 15.7 J/cm^2^, irradiance 17.4 mW/cm^2^ for 15 min). For dye-loaded PLGA NPs, the concentrations refer to dyes incorporated into PLGA (from 2 µM to 5 µM) in PBS buffer, pH 5.5. Data are reported as an average of 3 independent experiments. *p* values: * *p* < 0.05, ** *p* < 0.01, *** *p* < 0.005, and **** *p* < 0.001.

In this case, the survival of bacteria after treatment was less than 10% (Figure 11), observing notable antimicrobial activity in such conditions. The behavior of free SQ and empty PLGA remained unchanged at this acidic pH, confirming the non-toxicity in dark conditions and, in the case of empty PLGA, also after irradiation.

## 4. Conclusions

A bromine-substituted indolenine-based squaraine (BrSQ), already proposed as an excellent photosensitizing agent (PS) in Photodynamic Therapy (PDT) towards two different tumor cell lines, was selected to be considered as a potential candidate also for Antimicrobial Photodynamic Therapy (aPDT). However, its tendency to self-aggregate and its poor solubility in biological media still limit its wider applicability. To overcome these issues, our group proposed the incorporation of this squaraine dye into poly lactic-co-glycolic acid nanoparticles (PLGA NPs).

Among the different methods reported in the literature for the incorporation of hydrophobic PS into PLGA NPs, single emulsion and nanoprecipitation were selected and optimized with a statistical multivariate design, such as the design of experiment (DoE), in order to provide the maximum information while requiring a minimum number of experiments. Two different DoEs, one for each method, were prepared, altering various formulation parameters, i.e., type of solvent, solvent ratio, concentration of PLGA, stabilizer and dye, and sonication power and time, and assessing the size, ζ-potential, yield, and encapsulation efficiency. Based on the DoE results, the nanoprecipitation method (DoE-1) appeared not suitable for the synthesis of BrSQ-loaded PLGA nanoparticles. On the other hand, the single emulsion method (DoE-2) showed the optimal results in terms of encapsulation efficiency (EE), size, and yields. Concerning the size and ζ-potential of PLGA nanoparticles, no significant factors were identified to influence the two responses, with size ranges from 137–187 nm, while the ζ-potentials were found from −10 mV to −35 mV, all suitable values for NPs for biomedical application. The EE values obtained with DoE-2 range from 4% to 76%, resulting in being affected by different parameters with the following trend: sonication time > PLGA type > PLGA quantity > solvent type > stabilizer. Lastly, the measured yields exhibited values ranging from 6% to 85%, mainly affected by stabilizer > sonication time > solvent type > PLGA quantity.

Based on the DoE response, the formulation number 6 obtained by DoE-2 (PLGA amount: 70 mg; PLGA type: 65:35; stabilizer amount: 1.5 wt%; O/A ratio: 0.3; dye amount: 1.5 mg; solvent type: DCM; sonication time: 0.5 min; sonication power: 240W) was selected for further characterizations and for the evaluation of aPDT. The in vitro ROS generation of BrSQ and BrSQ-PLGA was evaluated, showing a fast ROS production with a minimal decrease for the PLGA-loaded NPs, probably due to the polymer matrix that scatters the light decreasing the light dose. Finally, the photodynamic effect of BrSQ and BrSQ-PLGA NPs against representative Gram-positive *Staphylococcus aureus* (*S. aureus*) bacteria was investigated under different conditions (i.e., dye concentration, irradiation time, irradiation power, and pH of the medium).

The higher aPDT effect was finally observed, keeping the following set-up: higher dye concentration (2, 3, and 5 µM), irradiance of 17.4 mW/cm^2^, and fluence of 15.7 J/cm^2^, performing the test at pH 5.5. In fact, the acidic pH promotes the swelling of the polymeric matrix, allowing the light to penetrate more deeply, reaching the loaded dye. In this case, the survival of bacteria after treatment was less than 10%. In all the cases, no toxicity in dark conditions was observed and, in the case of empty PLGA, also after irradiation.

Taken together, all these results highlight the benefits of incorporating BrSQ dye into PLGA NPs to optimize their photoactive properties. Moreover, since during a bacterial infection the pH can decrease up to 5.5–6, the in vitro antimicrobial results support the applicability of these stimuli-responsive nanosystems able to selectively kill bacteria as promising innovative PSs for aPDT applications.

Undoubtedly, for a possible future application, the antimicrobial effect has to be increased up to at least 99.9% of bacteria inactivation. The work reported in this manuscript constitutes the basic research, which is essential as a starting point prior to moving to future in vivo evaluation regarding biocompatibility assays and optimization of the antimicrobial activity (nanoformulation dosage, administration route, targeting, etc.).

## Figures and Tables

**Table 1:** Experimental variables and their relative ranges of investigation.

Variables	Abbreviation	Unit	Range (Level)	
			−1	+1
PLGA quantity	PLGAq	mg	30	70
PLGA type	PLGAt	-	65:35	50:50
Stabilizer quantity	Stab	%	0.5	1.5
Organic/aqueous phase ratio	O/A	-	0.3	0.7
Dye quantity	Dye	mg	0.5	1.5
Solvent type	Solv	-	Acetone ^1^, DCM ^2^	Acetonitrile ^1^, chloroform ^2^
Sonication time	Stm	min	0.5	3.5
Sonication power output	Spw	W	120	240

^1^ DoE-1 with nanoprecipitation method; ^2^ DoE-2 with single emulsion method.

## Data Availability

Data are contained within the article.
